# Targeted pharmacological treatment of autism spectrum disorders: fragile X and Rett syndromes

**DOI:** 10.3389/fncel.2015.00055

**Published:** 2015-02-26

**Authors:** Hansen Wang, Sandipan Pati, Lucas Pozzo-Miller, Laurie C. Doering

**Affiliations:** ^1^Faculty of Medicine, University of Toronto, 1 King’s College CircleToronto, ON, Canada; ^2^Department of Neurology, Epilepsy Division, The University of Alabama at BirminghamBirmingham, AL, USA; ^3^Department of Neurobiology, Civitan International Research Center, The University of Alabama at BirminghamBirmingham, AL, USA; ^4^Faculty of Health Sciences, Department of Pathology and Molecular Medicine, McMaster UniversityHamilton, ON, Canada

**Keywords:** fragile X syndrome, Rett syndrome, autism spectrum disorders, pharmacotherapy, treatment, synaptic deficits, FMRP, MeCP2

## Abstract

Autism spectrum disorders (ASDs) are genetically and clinically heterogeneous and lack effective medications to treat their core symptoms. Studies of syndromic ASDs caused by single gene mutations have provided insights into the pathophysiology of autism. Fragile X and Rett syndromes belong to the syndromic ASDs in which preclinical studies have identified rational targets for drug therapies focused on correcting underlying neural dysfunction. These preclinical discoveries are increasingly translating into exciting human clinical trials. Since there are significant molecular and neurobiological overlaps among ASDs, targeted treatments developed for fragile X and Rett syndromes may be helpful for autism of different etiologies. Here, we review the targeted pharmacological treatment of fragile X and Rett syndromes and discuss related issues in both preclinical studies and clinical trials of potential therapies for the diseases.

## Introduction

Autism spectrum disorders (ASDs) encompass a group of neurodevelopmental disorders which are of different etiologies and characterized by impairments in socialization and communication, abnormalities in language development, restricted interests, and repetitive and stereotyped behaviors (Mefford et al., 2012; Zoghbi and Bear, 2012; Murdoch and State, 2013; Anagnostou et al., 2014b; Lai et al., 2014). These disorders are estimated to affect approximately 1% of the population. The genetic causes of ASDs show a high degree of heterogeneity, with hundreds of ASD-associated genes now identified (Mefford et al., 2012; Huguet et al., 2013; Murdoch and State, 2013; Lai et al., 2014; Ronemus et al., 2014). However, recent studies suggest that these ASD genes may functionally converge into a relatively smaller subset of cellular and biochemical pathways affecting distinct neuronal functions (Auerbach et al., 2011; Zoghbi and Bear, 2012; Ebert and Greenberg, 2013; Doll and Broadie, 2014; Krumm et al., 2014). In a subset of ASDs, such as the syndromic ASD fragile X syndrome, Rett syndrome (RTT) and tuberous sclerosis, mutations of genes have been found to be related to synaptic function, suggesting that abnormal neuronal homeostasis is a risk factor for ASD (Auerbach et al., 2011; Santoro et al., 2012; Zoghbi and Bear, 2012; Ebert and Greenberg, 2013; Banerjee et al., 2014). This subset of ASDs belong to a larger group of neurological conditions called “synaptopathies”, which refer to disorders with altered synaptic function and/or morphology as primary neuropathology (Auerbach et al., 2011; Zoghbi and Bear, 2012; Krumm et al., 2014). The functional convergence on particular signaling pathways and the shared synaptopathology of ASDs have raised the hope that similar therapeutic strategies may be effective for different forms of ASDs which are related, but genetically distinct (Zoghbi and Bear, 2012; Delorme et al., 2013; Wang and Doering, 2013).

Fragile X and Rett syndromes are two of the most widely and intensively studied monogenetic ASDs (Santoro et al., 2012; Zoghbi and Bear, 2012; Castro et al., 2013; Chapleau et al., 2013a; Banerjee et al., 2014). Numerous studies have investigated the possibility of treating fragile X and Rett syndromes in their relative animal models. Strategies to alleviate abnormal phenotypes include genetic manipulation, cellular therapy, pharmacological intervention and environmental stimulation (Wang and Doering, 2012; Zoghbi and Bear, 2012; Castro et al., 2013; Chapleau et al., 2013a; Delorme et al., 2013; Ebert and Greenberg, 2013). Most encouraging, some of these fundamental studies have led to the development of drugs that are in clinical trials.

In this review, we summarize the potential therapeutic targets and relative pharmacological interventions in fragile X and Rett syndromes. The challenges in preclinical studies and clinical trials, and the implications of these targeted pharmacological treatments for other ASDs and related neurodevelopmental disorders are also discussed.

## Targeted pharmacotherapy for fragile X syndrome

Fragile X syndrome is one of the most common genetic causes of intellectual disability and autism. It is mostly caused by the mutation of the trinucleotide CGG expansion in the 5′-untranslated region of the fragile X mental retardation (*FMR1*) gene, which eventually leads to the absence of its protein product, fragile X mental retardation protein (FMRP) (Garber et al., 2008; Bhakar et al., 2012; Santoro et al., 2012; Hagerman et al., 2014). FMRP is ubiquitous with the most abundance in the central nervous system (Santoro et al., 2012; Wang et al., 2012a; Sidorov et al., 2013) and exists in both neurons and glial cells (Wang et al., 2004; Pacey and Doering, 2007).

FMRP, a RNA binding protein, binds its mRNA targets and regulates the transport and translation of those mRNAs (Bear et al., 2004; Penagarikano et al., 2007; Bhakar et al., 2012; Santoro et al., 2012; Wang et al., 2012a; Sidorov et al., 2013; Abekhoukh and Bardoni, 2014). The mRNA targets of FMRP encode pre- and post-synaptic proteins and some of these proteins are implicated in other ASDs, suggesting a molecular overlap between fragile X syndrome and other neurodevelopmental disorders (Bhakar et al., 2012; Wang et al., 2012a; Sidorov et al., 2013). FMRP normally acts as a translational repressor, especially at synapses. Its absence has profound consequences on neural development and synaptic plasticity. Alterations in synaptic structure and function are believed to underlie the fragile X symptoms. Dysregulated protein synthesis is central to fragile X synaptopathy (Bear et al., 2004; Penagarikano et al., 2007; Bassell and Warren, 2008; Wang et al., 2010a; Bhakar et al., 2012; Santoro et al., 2012; Sidorov et al., 2013).

**Figure 1:**
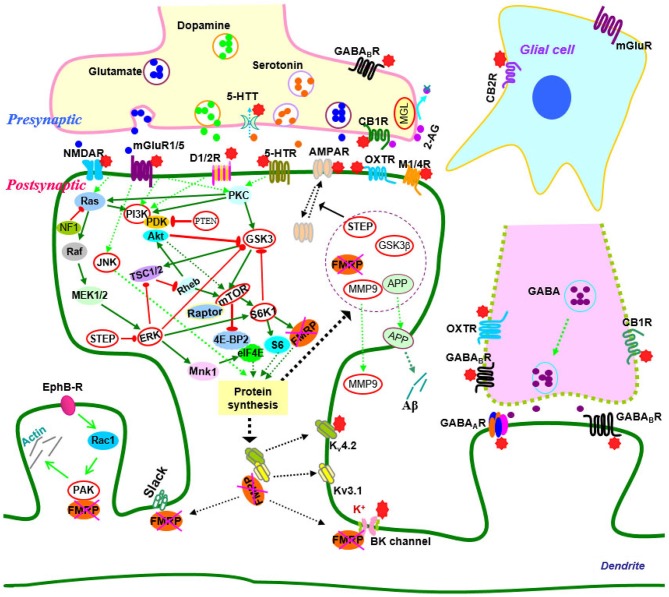
**Signaling molecules and pathways in the neurobiology of fragile X syndrome**. In signaling pathways, arrows indicate positive (green) or inhibitory (red) consequence on downstream components, but they do not necessarily represent direct interactions. Potential therapeutic targets which have been validated by genetic or pharmacological manipulation are indicated by red stars or highlighted by red circles. Abbreviations: 2-AG, 2-arachidonoyl-sn-glycerol; 4E-BP2, eIF4E-binding protein 2; 5-HT, serotonin; 5-HTR, serotonin receptors; 5-HTT, serotonin transporters; Akt (PKB), protein kinase B; AMPAR, α-amino-3-hydroxyl-4-isoxazole propionic acid receptors; APP, amyloid precursor protein; CB1(2)R, cannabinoid receptor 1(2); EphB-R, EphB receptors; ERK, extracellular signal related kinase; FMRP, fragile X mental retardation protein; GABA, gamma aminobutyric acid; GABA_A(B)_R, GABA_A(B)_ receptors; GSK3, glycogen synthase kinase-3; JNK, c-Jun N-terminal kinase; M1/4R, muscarinic acetylcholine receptor 1/4; MEK, mitogen-activated protein kinase/ERK kinase; MGL, monoacylglycerol lipase; mGluR, metabotropic glutamate receptor; MMP9, matrix metalloproteinase 9; MSK, mitogen and stress-activated protein kinase; mTOR, mammalian target of rapamycin; NF1, neurofibromatosis 1; NMDAR, N-methyl-d-aspartate receptors; OXTR, oxytocin receptor; PAK, p21-activated kinase; PI3K, phosphatidylinositol 3-kinase; PKC, protein kinase C; PP2A, protein phosphatase 2A; PTEN, Phosphatase and tensin homolog; Raptor, regulatory-associated protein of mTOR; RSK, p90 ribosomal S6 kinase; S6K1, p70 ribosomal kinase 1; STEP, striatal-enriched protein tyrosine phosphatase; TSC1/2, tuberous sclerosis complex 1/2.

Over the past two decades, efforts have been made to elucidate the molecular and cellular events that give rise to synaptic dysfunction in fragile X syndrome. Studies in animal models have revealed defects in multiple neurotransmitter systems and relative signaling pathways/molecules that are responsible for fragile X synaptopathy (Wang et al., 2010b; Bhakar et al., 2012; Gross et al., 2012; Santoro et al., 2012; Zoghbi and Bear, 2012; Delorme et al., 2013; Ebert and Greenberg, 2013). Advances in neurobiology of fragile X syndrome have led to the development of therapeutic agents that target the underlying mechanisms of the disease (Summarized in Figure 1; Tables 1, 2).

**Table 1:** **Therapeutic targets and relative drugs in preclinical studies of fragile X syndrome**.

Targets		Drugs	Action
*Neurotransmitter receptors or transporters*	mGluR5	MPEP, CTEC, fenobam, AFQ056	Antagonists
	mGluR1	JNJ16259685, LY367385	Antagonists
	GABA_A_ receptor	THIP, ganaxolone	Agonists
	GABA_B_ receptor	Baclofen, arbaclofen (STX209)	Agonists
	5-HT_7_ receptor	8-OH-DPAT	Agonist
	5-HT_2B_ receptor	BW723C86	Agonist
	5-HT_2A_ receptor	***α***Me-5HT, MDL11939	Antagonists
	5-HTT	Fluoxetine	5-HT reuptake inhibitor
	Dopamine D1 receptor	SKF81297	Agonist
	CB1R	Rimonabant	Antagonist
	CB2R	AM630	Antagonist
	MGL	JZL184	Inhibitor
	M1R	Dicyclomine	Antagonist
	M4R	Tropicamide	Antagonist
	NMDAR	Memantine	Antagonist
*Protein kinases or other enzymes*	ERK	SL327, U0126, lovastatin	Inhibitors
	JNK	SP600125	Inhibitor
	PI3K	LY294002, TGX-221	Inhibitors
	PTEN	BpV	Inhibitor
	mTOR	Temsirolimus, rapamycin, LY294002, TGX221	Inhibitors
	GSK3	Lithium, SB216763 TDZD-8, YP0.7	Inhibitors
	MMP9	Minocycline	Inhibitor
	PAK	FRAX486	Inhibitor
*Ion channels*	Kv4 channel	HpTx2	Blocker
	BK channel	BMS-204352	Opener

**Table 2:** **Drugs in clinical trials of fragile X syndrome**.

Drugs	Action	Study designs	Publications
Fenobam	mGluR5 antagonist	Open label pilot study; single dose	Berry-Kravis et al. (2009)
AFQ056	mGluR5 antagonist	Randomized, double-blind, two treatment, two-period crossover study	Jacquemont et al. (2011)
RO4917523	mGluR5 antagonist	Randomized, double-blinded, placebo-controlled study	Discontinued due to negative results^†^
Ganaxolone	GABA_A_ receptor agonist	Double-blind, controlled trial	www.clinicaltrials.gov
Acamprosate	GABA receptor agonist	Open-label, uncontrolled trial	Erickson et al. (2010, 2013)
Arbaclofen (STX209)	GABA_B_ receptor agonist	Randomized, double-blind, placebo-controlled study	Berry-Kravis et al. (2012)
Oxytocin	OXTR agonist	Double-blind, placebo-controlled study	Hall et al. (2012)
CX516	AMPAR positive modulator	Randomized, double-blind, placebo-controlled study	Berry-Kravis et al. (2006)
Memantine	NMDAR antagonist	Open-label study	Erickson et al. (2009)
Lovastatin	ERK inhibitor	Open-label study	Çaku et al. (2014)
Lithium	GSK3 inhibitor	Open-label study	Berry-Kravis et al. (2008)
Minocycline	MMP9 inhibitor	Open-label add-on pilot trial	Paribello et al. (2010)
		Randomized, double-blind, placebo-controlled and crossover trial	Leigh et al. (2013)

^†^www.fragilex.org

### Targeting neurotransmitter/neuromodulator systems

#### Metabotropic glutamate receptors

Metabotropic glutamate receptors (mGluRs) play important roles in synaptic plasticity, learning and memory (Bortolotto et al., 1999; Bear et al., 2004; Wang et al., 2008a; Wang and Zhuo, 2012; Mukherjee and Manahan-Vaughan, 2013). Activation of group 1 mGluRs (mGluR1s and mGluR5s) leads to local translation of pre-existing mRNAs and triggers long-term depression (LTD) (Weiler and Greenough, 1993; Bortolotto et al., 1999; Raymond et al., 2000; Penagarikano et al., 2007; Ronesi and Huber, 2008a; Upreti et al., 2013). FMRP is one of these newly synthesized proteins and serves as a repressor of the translation of other synaptic mRNAs that encode LTD proteins such as activity-regulated cytoskeleton-associated protein (Arc), microtubule associated protein (MAP) 1B and Striatal-enriched protein tyrosine phosphatase (STEP), which mediate a-amino-3-hydroxyl-4-isoxazole propionic acid receptor (AMPAR) internalization and LTD stabilization (Bhakar et al., 2012; Santoro et al., 2012; Wang et al., 2012a; Darnell and Klann, 2013; Sidorov et al., 2013). In fragile X conditions, protein synthesis is elevated and mGluR-LTD in the hippocampus is exaggerated in the absence of FMRP; the enhanced mGluR-LTD is no longer protein synthesis dependent as a result of increased basal protein synthesis. The mGluR theory of fragile X syndrome thus postulates that the core symptoms of the disease result from exaggerated group 1 mGluR mediated signaling including mGluR-LTD (Bear et al., 2004; Garber et al., 2008; Bhakar et al., 2012; Santoro et al., 2012; Sidorov et al., 2013).

The mGluR theory and its therapeutic significance have been validated in both genetic and pharmacological rescue studies (Dölen and Bear, 2008; Hays et al., 2011; Thomas et al., 2011; Gandhi et al., 2014; Michalon et al., 2014; Pop et al., 2014). In *Fmr1* knockout mice, the mGluR5 antagonist 2-methyl-6-(phenylethynyl)-pyridine (MPEP) stabilized hippocampal protein synthesis, increased the density or rescued the morphology of hippocampal dendritic spines, corrected altered brain network function, reduced audiogenic seizures and repetitive and/or perseverative behaviors (marble burying), rescued the deficits in prepulse inhibition of startle response, and improved the maze and motor learning (Yan et al., 2005; de Vrij et al., 2008; Hays et al., 2011; Thomas et al., 2012; Gandhi et al., 2014). Other mGluR5 antagonists tested in fragile X animal models include CTEC, fenobam and AFQ056. Acute treatment with CTEC corrected the elevated hippocampal LTD, protein synthesis, and audiogenic seizures; chronic treatment rescued cognitive deficits, auditory hypersensitivity, aberrant dendritic spine density, overactive extracellular signal regulated kinase (ERK) and mammalian target of rapamycin (mTOR) signaling, and partially corrected macroorchidism in adult fragile X mice (Michalon et al., 2012). Chronic CTEP treatment also corrected learning deficit in the inhibitory avoidance and extinction test, and partially normalized altered local brain activity in these animals (Michalon et al., 2014). Fenobam reversed some synaptic alterations in the cortex (Wang et al., 2014) and corrected deficits in associative motor learning and avoidance behaviors in fragile X mice (Vinueza Veloz et al., 2012). AFQ056 was found to be able to correct aberrant hippocampal dendritic spine morphology (Levenga et al., 2011; Pop et al., 2014), and rescue deficits in prepulse inhibition of acoustic startle response and abnormal social behaviors (Levenga et al., 2011; Gantois et al., 2013) in *Fmr1* knockout mice. Treatment with mGluR1 antagonists (JNJ16259685 or LY367385) decreased repetitive and/or perseverative behaviors (Thomas et al., 2012), and rescued dysregulated synaptic protein synthesis in fragile X mice (Gross et al., 2010; Guo et al., 2012). These preclinical studies have been paving the way for treatments with mGluR antagonists in humans.

Fenobam, the first mGluR antagonist used in patients, showed beneficial effects, such as reduced anxiety and hyperarousal, improved prepulse inhibition of startle, and better accuracy on a continuous performance task, in adults with fragile X syndrome (Berry-Kravis et al., 2009). The pharmacokinetics and side effects of fenobam are currently tested in adult healthy volunteers.^1^ AFQ056 also showed improvements in inappropriate speech, stereotypic behavior, and hyperactivity and efficacy in adult patients with fully methylated *FMR1* (Jacquemont et al., 2011). However, the mGluR5 negative allosteric modulator RG7090 (RO4917523) in fragile X was recently discontinued by Roche due to negative phase II clinical study results from fragile X patients.^2^ More drugs targeting mGluRs with higher efficacy and better safety need to be developed in the future (Berry-Kravis, 2014; Hagerman et al., 2014).

#### GABAergic receptors

The γ-aminobutyric acid (GABA) is the main inhibitory neurotransmitter in brain and signals through GABA_A_ and GABA_B_ receptors (Ben-Ari et al., 2012; Gassmann and Bettler, 2012; Sigel and Steinmann, 2012). The deficiencies in GABA receptor expression and GABA receptor-mediated inhibition have been demonstrated in fragile X animal models, linking GABA receptors to fragile X phenotypes and leading to the hypothesis that fragile X syndrome may result from an imbalance between excitation and inhibition, and increasing inhibition may ameliorate some fragile X pathophysiologies, including dysregulated protein synthesis (D’Hulst et al., 2006, 2009; Chang et al., 2008; Pacey et al., 2009; Olmos-Serrano et al., 2010; Paluszkiewicz et al., 2011; He et al., 2014). The potential of GABA receptors as a therapeutic target for fragile X syndrome has been validated in animal models. Treatment with a selective GABA_A_ receptor agonist THIP (gaboxadol) corrected neuronal hyperexcitability in the amygdala (Olmos-Serrano et al., 2010), and attenuated hyperactivity and deficits in prepulse inhibition of the acoustic startle response of fragile X mice (Olmos-Serrano et al., 2011). Treatment with ganaxolone, a positive allosteric modulator of GABA_A_ receptors, could prevent audiogenic seizures in *Fmr1* knockout mice (Heulens et al., 2012). The GABA_B_ receptor agonist baclofen reduced locomotor activity and hyperactivity (Zupan and Toth, 2008), and ameliorated audiogenic seizure susceptibility of *Fmr1* knockout mice (Pacey et al., 2009, 2011). Notably, arbaclofen (STX209), the R-isomer of baclofen, was also able to attenuate audiogenic seizures in fragile X mice (Henderson et al., 2012). In addition, arbaclofen corrected dysregulated protein synthesis in the hippocampus, restored the elevated AMPA receptor internalization and the increased spine density, and thus corrected synaptic abnormalities which are central to fragile X pathophysiologies, suggesting that arbaclofen could be potentially used to treat the core symptoms of fragile X patients (Henderson et al., 2012).

A large clinical trial with ganaxolone is currently ongoing in children and adolescents with fragile X syndrome between 6 to 17 years of age (Wijetunge et al., 2013; Berry-Kravis, 2014; Hagerman et al., 2014).^3^ A controlled trial of arbaclofen in 63 fragile X patients between 6 to 40 years of age has revealed significant beneficial treatment effects, such as improvements in socialization and social avoidance scores, particularly in those with more severe social impairments, suggesting that GABA_B_ receptor agonists have potential to improve social function and behaviors in fragile X patients (Berry-Kravis et al., 2012). However, this clinical trial for arbaclofen failed to fulfill its initial endpoints and had to be terminated by Seaside Therapeutics due to resource limitations.^4^ Acamprosate, with agonist properties on both GABA_A_ and GABA_B_ receptors, was well tolerated and was found to significantly improve social behavior and reduce inattention/hyperactivity in 12 fragile X children aged 6–17 years in a prospective open-label 10-week trial. Additionally, an increase in brain-derived neurotrophic factor (BDNF) in blood was observed with treatment and might become a useful biomarker for future clinical studies (Erickson et al., 2010, 2013). A double-blind, placebo-controlled study will be needed to further assess the effect of acamprosate. As a whole, it is hopeful that some of the GABA receptor agonists may eventually become clinically applicable.

#### Serotonin receptors/transporters

The serotonin (5-Hydroxytryptamine, 5-HT) system is involved in various brain functions, including synaptic plasticity, learning and memory (Matthys et al., 2011; Lesch and Waider, 2012; Gellynck et al., 2013). It has been shown that 5-HT7 receptor (agonist 8-OH-DPAT) activation could reverse mGluR-induced AMPA receptor internalization and correct excessive mGluR-LTD in hippocampal neurons of *Fmr1* knockout mice, suggesting that 5-HT receptors may represent a novel therapeutic target for fragile X syndrome (Costa et al., 2012). Interestingly, a recent study has demonstrated that compounds activating 5-HT2B receptors or inhibiting 5-HT2A receptors could enhance phosphoinositide 3-kinase (PI3K) signaling transduction and AMPA GluR1 dependent synaptic plasticity, and restore learning in *Fmr1* knockout mice, further linking 5-HT receptors to fragile X syndrome (Lim et al., 2014).

The alteration of 5-HT and its transporters have been known to associate with autism (Chugani, 2002; Devlin et al., 2005; Prasad et al., 2009; Veenstra-VanderWeele et al., 2012; Harrington et al., 2013). 5-HT transporters are the main target for widely used antidepressant agents including fluoxetine (Wong et al., 2005). Fluoxetine binds to 5-HT transporters and blocks 5-HT uptake from the synaptic cleft into presynaptic vesicles in the central nervous system (Wong et al., 2005; Tavoulari et al., 2009). Studies have suggested that fluoxetine may be beneficial to individuals with autism (Hollander et al., 2005, 2012; Kolevzon et al., 2006). Treatment with fluoxetine has shown some effect in fragile X mice and patients (Hagerman et al., 1999; Uutela et al., 2014). However, fluoxetine has some possible side effects in clinic, such as mood changes, agitation, restlessness, and aggression, and may not be suitable to all individuals with fragile X syndrome and other ASDs (Wernicke, 2004; Kolevzon et al., 2006; Uutela et al., 2014).

#### Dopamine receptors

Dopamine plays critical roles in synaptic plasticity, cognitive functioning and neuropsychiatic pathologies (Jay, 2003; Seamans and Yang, 2004; Surmeier et al., 2009; Cerovic et al., 2013). Deficits in the dopamine system have been demonstrated in both fragile X animal models and patients (Roberts et al., 2005; Wang et al., 2008b; Weinshenker and Warren, 2008; Fulks et al., 2010; Paul et al., 2013; Rogers et al., 2013). Electrophysiological studies have found that dopaminergic modulation of synaptic transmission and potentiation are impaired in fragile X mice (Wang et al., 2008b; Paul et al., 2013). Biochemical studies have further revealed that dopamine D1 receptor mediated synapse-associated protein synthesis, AMPA GluR1 receptor surface expression and subsequent internalization are defective in these mice (Wang et al., 2008b, 2010a). Application of the D1 receptor agonist and/or D2 receptor antagonist could promote PI3K signaling and AMPA GluR1 receptor delivery, and improve learning behaviors in* Fmr1* knockout mice (Lim et al., 2014). The dopamine D1 receptor agonist also partially rescued the hyperactivity and enhanced motor function of fragile X mice (Wang et al., 2008b). These findings suggest that dopamine receptors could be a potential target for effectively treating cognitive impairment associated with fragile X syndrome.

#### Cannabinoid receptors

The endocannabinoids N-arachidonoyl ethanolamine and 2- arachidonoyl glycerol (2-AG) activate cannabinoid receptors (CB1R and CB2R), and modulate synaptic plasticity and cognitive function (Chevaleyre et al., 2006; Heifets and Castillo, 2009; Oudin et al., 2011). Alterations in endocannabinoid signaling contribute to cognitive dysfunction associated with fragile X syndrome. Various abnormalities in endocannabinoid signaling, such as CB1R-driven long-term regulation of synaptic strength due to mGluR5 activation, have been observed in several brain areas of *Fmr1* knockout mice (Maccarrone et al., 2010; Zhang and Alger, 2010; Busquets-Garcia et al., 2013). FMRP exerts a regulatory control over the endocannabinoid system at central synapses (Maccarrone et al., 2010). Loss of FMRP affects endocannabinoid signaling, possibly through local 2-AG production (Straiker et al., 2013). In fragile X mice, the linkage between mGluR5 and the 2-AG producing enzyme, diacylglycerol lipase-α (DGL-α), is disrupted and mGluR5-dependent 2-AG formation is compromised, leading to impairment of endocannabinoid-mediated LTD in the ventral striatum and prefrontal cortex (Jung et al., 2012).

Pharmacological enhancement of 2-AG signaling by inhibiting 2-AG-deactivating enzyme monoacylglycerol lipase (MGL) with JZL184, normalized this synaptic defect and corrected behavioral abnormalities (hyperactivity and abnormal anxiety) in fragile X mice (Jung et al., 2012). Interestingly, dampening of endocannabinoid signaling through pharmacological or genetic approaches also benefit these mice. CB1R blockade with rimonabant or genetic reduction of CB1R, normalized cognitive impairment, nociceptive desensitization, susceptibility to audiogenic seizures, overactivated mTOR signaling and altered spine morphology in the male *Fmr1* knockout (*Fmr1*(-/y)) mice, whereas CB2R antagonism with AM630 normalized anxiolytic-like behaviors in those mice (Busquets-Garcia et al., 2013). These studies thus demonstrate that targeting endocannabinoid signaling might provide a new therapeutic strategy for fragile X syndrome. Further investigation is needed to clarify the therapeutic value of this potential target.

#### Muscarinic acetylcholine receptors

The G-protein coupled muscarinic acetylcholine receptors (mAChRs, subtypes M1–M5) are widely expressed in the central nervous system and mediate the metabotropic actions of acetylcholine (Volpicelli and Levey, 2004; Picciotto et al., 2012). M1 mAChR activation facilitates synaptic plasticity, learning and memory. Activation of M1 mAChRs stimulates synthesis of synaptic proteins including FMRP, and induces protein synthesis dependent LTD similar to group 1 mGluR-LTD (McCoy and McMahon, 2007; Volk et al., 2007). In the absence of FMRP, hippocampal M1 mAChR-dependent LTD is enhanced, indicating overactive mAChR signaling in* Fmr1* knockout mice (Volk et al., 2007). Genetic reduction of M4 mAChRs corrected the analgesic response and partly rescued the acoustic startle response in *Fmr1* knockout mice (Veeraragavan et al., 2012). These studies suggest new therapeutic strategies for using mAChR antagonists in fragile X syndrome.

The subtype selective mAChR modulators, including the M1 receptor antagonist dicyclomine and M4 receptor antagonist tropicamide, have been tested in the fragile X mouse model. The M1 receptor antagonist dicyclomine decreased repetitive and/or perseverative behavior (marble burying) and reduced susceptibility to audiogenic seizures in *Fmr1* knockout mice (Veeraragavan et al., 2011b). Similarly, the M4 receptor antagonist tropicamide also attenuated audiogenic seizure susceptibility in *Fmr1* knockout mice (Veeraragavan et al., 2011a). Noteworthy, treatment with tropicamide reduced repetitive and/or perseverative behaviors, improved performance in the passive avoidance task in both wild-type and fragile X mice, and reduced audiogenic seizures in fragile X mice (Veeraragavan et al., 2011a). These studies indicate that pharmacological inhibition of mAChRs modulates specific behavioral responses and further support these receptors as therapeutic targets for fragile X syndrome.

#### Oxytocin receptors

Oxytocin acts as a neuromodulator through its receptors in various brain areas and regulates social cognition and behaviors (Neumann, 2008; Meyer-Lindenberg et al., 2011; Neumann and Landgraf, 2012; Knobloch and Grinevich, 2014). It is emerging as a target for treatment of anxiety and depression-related diseases or social dysfunction including autism (Neumann, 2008; Meyer-Lindenberg et al., 2011; Neumann and Landgraf, 2012; Anagnostou et al., 2014a; Preti et al., 2014). The perinatal excitatory-to-inhibitory shift of GABA is mediated by oxytocin receptors. Oxytocin-mediated GABA inhibition during delivery could attenuate autism pathogenesis in rodent offsprings. Application of the oxytocin receptor antagonist SSR126768A in naïve mothers could produce offspring which have the electrophysiological and behavioral autistic-like features (Tyzio et al., 2014). In fragile X mice, oxytocin is reduced in some brain regions and the oxytocin-mediated neuroprotective GABA excitatory-inhibitory shift during delivery is absent (Tyzio et al., 2014). This study thus indicates the importance of the oxytocin system in the pathogenesis of autism and fragile X sydrome.

The clinical actions of oxytocin have been validated in ASD patients, providing preliminary evidence that oxytocin is able to enhance brain function and improve social behaviors in autistic patients (Andari et al., 2010; Domes et al., 2013; Gordon et al., 2013; Anagnostou et al., 2014a; Preti et al., 2014; Scheele et al., 2014; Watanabe et al., 2014). Intranasal administration of oxytocin could ameliorate symptoms of social anxiety in children with fragile X syndrome (Hall et al., 2012). The double-blind placebo-controlled studies of oxytocin will be needed for further validation of its effect on fragile X patients.

#### AMPA receptors

The surface expression and synaptic delivery of AMPA receptors (GluR1) is impaired in *Fmr1* knockout mice, altering signal transmission and synaptic plasticity (Nakamoto et al., 2007; Hu et al., 2008; Suvrathan et al., 2010; Wang et al., 2010a). AMPA receptor modulators have been tested in fragile X patients. CX516 is a positive allosteric modulator that potentiates glutamate activation with the final outcome of strengthening synapses (O’Neill et al., 2004; O’Neill and Witkin, 2007). In a double-blind placebo-controlled trial in adult patients with fragile X syndrome, 4-week treatment with CX516 produced no significant improvement in cognitive or behavioral measures (Berry-Kravis et al., 2006). The failure could be due to the potency of CX516 or its dosage which may be inadequate for a therapeutic effect. It is thus unclear whether modulation of AMPA receptors is a feasible therapeutic strategy for treatment of fragile X syndrome.

#### NMDA receptors

The defects in NMDA receptors observed in the hippocampus, prefrontal cortex and other brain areas of fragile X mice are believed to contribute to fragile X phenotypes (Pilpel et al., 2009; Suvrathan et al., 2010; Krueger et al., 2011; Yun and Trommer, 2011; Eadie et al., 2012; Gocel and Larson, 2012). Memantine is an uncompetitive NMDA receptor antagonist and has been shown to improve language function and social behavior in autistic patients (Erickson and Chambers, 2006; Chez et al., 2007; Niederhofer, 2007). A study in cultured cerebellar granule cells from *Fmr1* knockout mice suggested that memantine may exert therapeutic capacity for fragile X syndrome through a stimulatory effect on dendritic spine maturation and excitatory synapse formation (Wei et al., 2012). Although a pilot study showed that memantine was modestly effective in several patients with fragile X syndrome, a systematic clinical study is needed to further evaluate its effectiveness (Erickson et al., 2009).

### Targeting signaling pathways downstream of neurotransmitter receptors

Studies in fragile X animal models have revealed defects in intracellular signaling pathways [Mitogen-activated protein kinases (MAPKs), PI3K, mTOR and glycogen synthase kinase-3 (GSK3)] which could be downstream of neurotransmitter receptors such as glutamate, GABA, endocannabinoid, 5-HT, dopamine or mACh receptors. These signaling pathways may serve as therapeutic targets for fragile X syndrome (Figure 1; Tables 1, 2).

#### Mitogen-activated protein kinases

MAPKs are a family of serine/threonine protein kinases, including ERKs, p38 MAPKs, and c-Jun N-terminal kinase (JNK; Wang and Zhuo, 2012). The ERK1/2 pathway, which is activated by the Ras-mitogen-activated protein kinase/ERK kinase (MEK) and eventually leads to gene transcription or mRNA translation, plays critical roles in synaptic plasticity (Kelleher et al., 2004; Thomas and Huganir, 2004; Wiegert and Bading, 2011; Wang and Zhuo, 2012). A number of studies have investigated ERK signaling under basal conditions or upon mGluR-induction using brain tissues from fragile X animal models or patients (Kim et al., 2008; Weng et al., 2008; Wang et al., 2012b; Curia et al., 2013). Although these studies sometimes have presented conflicting results, most of them have shown that the ERK1/2 pathway is altered in fragile X conditions, suggesting that the ERK pathway is likely to have translational implications for fragile X syndrome (Weng et al., 2008; Wang et al., 2012b; Curia et al., 2013). Indeed, treatment with MEK1/2 inhibitor SL327 could prevent audiogenic seizures in *Fmr1* knockout mice (Wang et al., 2012b). Inhibition of ERK1/2 with the MEK1/2-ERK1/2 inhibitor U0126 also reduced elevated protein synthesis in hippocampus of fragile X mice (Osterweil et al., 2010). These findings further support that ERK1/2 pathway and the neurotransmitter systems that stimulate ERK1/2 may represent additional therapeutic targets for fragile X syndrome. Interestingly, statins, drug widely prescribed to treat hypercholesterolemia, have shown potentials for treating fragile X syndrome. Lovastatin corrected the excess protein synthesis and the exaggerated mGluR-LTD in the hippocampal slices of *Fmr1* knockout mice, attenuated hyperexcitability in visual cortex, and reduced audiogenic seizures in those mice (Osterweil et al., 2013). In a recent Phase I clinical trial, lovastatin was found to improve aberrant behaviors (social avoidance/unresponsiveness, stereotypy, hyperactivity and irritability) in majority (12/15) of fragile X patients after 12-weeks of treatment; the effect was significant after 4-week treatment at the lower dose, with further improvement during the 4–12 week treatment (Çaku et al., 2014). Although it was suggested that statins may alter membrane cholesterol and lipid rafts, thus modulating group I mGluRs or/and other neurotransmitter systems to correct fragile X phenotypes, the drugs might also exert the effects through direct inhibition of Ras–ERK activity (Kumari et al., 2013; Osterweil et al., 2013; Wang, 2014).

The JNK pathway is known to regulate mGluR-dependent gene transcription (Wang and Zhuo, 2012). A recent study has shown that JNK is essential for mGluR-dependent expression of FMRP target proteins. In addition, JNK activity is upregulated in synapses of *Fmr1* knockout mice, and inhibition of JNK with SP600125 decreased elevated postsynaptic protein synthesis in these mice, suggesting that JNK could be a key signaling downstream of mGluR in regulating FMRP-dependent protein synthesis and may provide a strategy to restore the deficits in fragile X syndrome (Schmit et al., 2013).

#### Phosphoinositide 3-kinase

PI3K transduces signals from cell surface receptors to the Akt/mTOR pathway and is essential for dendritic spine development and synaptic plasticity underlying learning and memory (Horwood et al., 2006; Hu et al., 2008; Cuesto et al., 2011). The PI3K catalytic subunit p110beta can be regulated by FMRP. Both p110beta level and PI3K activity are elevated and insensitive to mGluR stimulation in *Fmr1* knockout neurons, suggesting that dysregulated PI3K signaling may underlie synaptic deficits in fragile X syndrome (Gross et al., 2010; Gross and Bassell, 2014). PI3K inhibitor LY294002 corrected dysregulated synaptic protein synthesis, excess AMPA receptor internalization and the increased spine density in *Fmr1* knockout neurons, supporting PI3K as a potential therapeutic target for fragile X syndrome (Gross et al., 2010). Development of specific inhibitors for PI3K subunits may help to translate this strategy to patients since selective inhibition of the p110b-subunit with TGX-221 has been found to rescue excess protein synthesis in synaptoneurosomes from fragile X mice and in patient cells (Gross et al., 2010; Gross and Bassell, 2012, 2014). Conversely, one recent study showed that promoting PI3K signaling by dipotassium bisperoxo (5-hydroxypyridine-2-carboxyl)oxovanadate (BpV), a phosphatase and tensin homolog (PTEN) inhibitor, reversed deficits in both basal turnover and activity-mediated spine stabilization in hippocampal slices, restored defective long-term potentiation (LTP) mechanisms in slices and improved reversal learning in *Fmr1* knockout mice (Boda et al., 2014). Thus, the specific role of PI3K signaling in fragile X syndrome needs to be further investigated due to the complexity of its upstream cell surface receptors and interactions with other signaling pathways.

#### Mammalian target of rapamycin

The mTOR signaling cascade controls initiation of cap-dependent translation (Hay and Sonenberg, 2004; Narayanan et al., 2008; Hoeffer and Klann, 2010). Its downstream effector ribosomal protein S6 kinase (S6K1) is a regulator of translation initiation and elongation in cap-dependent protein synthesis (Narayanan et al., 2008; Hoeffer and Klann, 2010). S6K1 is a major kinase which phosphorylates and regulates FMRP following group 1 mGluR or dopamine D1 receptor activation (Narayanan et al., 2008; Wang et al., 2010a). The basal levels of mTOR phosphorylation and activity were found to be elevated in the hippocampus of *Fmr1* knockout mice (Sharma et al., 2010). In addition, group 1 mGluR activation of mTOR is absent is those mice (Ronesi and Huber, 2008b). The misregulation of mTOR signaling was also observed in fragile X patients (Hoeffer et al., 2012), suggesting that mTOR could be a target or biomarker for treatment of fragile X syndrome.

Treatment of *Fmr1* knockout mice with temsirolimus, an mTOR inhibitor, prevented object recognition memory deficits and reduced audiogenic seizure susceptibility in those mice (Busquets-Garcia et al., 2013). Elevated phosphorylation of translational control molecules and exaggerated protein synthesis in fragile X mice were corrected through the targeting of S6K1. Genetic deletion of S6K1 also prevented a broad range of fragile X phenotypes, including exaggerated translation, enhanced mGluR-LTD, abnormal dendritic spine morphology, several behavioral characteristics and peripheral features (weight gain and macroorchidism) (Bhattacharya et al., 2012). Notably, administration of the mTORC1 (mTOR complex 1) inhibitor rapamycin improved sociability in the BTBR mouse model of ASDs (Burket et al., 2014); targeting downstream mTOR signaling such as eukaryotic translation initiation factor 4E (eIF4E) also reversed autism (Gkogkas et al., 2013; Wang and Doering, 2013). These studies thus support that mTOR signaling represents a potential therapeutic target for fragile X syndrome and other ASDs.

#### Glycogen synthase kinase-3

GSK3 is a serine/threonine kinase that exists in two isoforms (GSK3α and GSK3β) and regulates many cellular processes through phosphorylation of their substrates. The activity of GSK3 itself is controlled by inhibitory serine phosphorylation induced by various intracellular pathways including PI3K/Akt and MEK/ERK that converge on GSK3 (Cohen and Goedert, 2004; Jope and Roh, 2006; Sugden et al., 2008). In *Fmr1* knockout mice, GSK3 phosphorylation was reduced in several brain regions resulting in elevated GSK3 signaling (Min et al., 2009; Mines et al., 2010; Yuskaitis et al., 2010). Lithium, a classical drug for psychiatric disorders, is a GSK3 inhibitor that both increases the inhibitory serine-phosphorylation of GSK3 and directly inhibits GSK3 activity (Chiu and Chuang, 2010; Mines and Jope, 2011). Since lithium has many other off-target effects including inhibition of inositol monophosphatase, more selective GSK3 inhibitors have been developed (Chiu and Chuang, 2010; Mines and Jope, 2011). Lithium, as well as the selective GSK3β inhibitor SB216763, was found to be able to correct mutant phenotypes (audiogenic seizure susceptibility and hyperactivity) of *Fmr1* knockout mice. Particularly, lithium remained effective with chronic administration (Min et al., 2009). Acute or chronic lithium treatment could increase inhibitory serine phosphorylation of GSK3 in mouse brain; chronic treatment ameliorated alterations in open-field activity, elevated plus-maze and passive avoidance and impaired cognition in fragile X mice (Yuskaitis et al., 2010; King and Jope, 2013). Chronic treatment with SB216763 corrected hippocampus-dependent learning deficits as well as defects in adult neurogenesis in the hippocampus of fragile x mice (Guo et al., 2012). The specific GSK3 inhibitors TDZD-8 and VP0.7 corrected impairments in hippocampus-related cognitive tasks. Furthermore, the improvements in behaviors correlated to the rescue of deficits in NMDA receptor dependent LTP in the hippocampus of *Fmr1* knockout mice as a result of GSK3 inhibition (Franklin et al., 2014). These studies thus support that targeting GSK3 may provide therapeutic benefits for fragile X syndrome.

In an open-label trial in 15 patients, the two month treatment with lithium was well-tolerated and had positive effects on behavior and adaptive skills in fragile X syndrome (Berry-Kravis et al., 2008). The placebo-controlled trials of lithium or other GSK3 inhibitors in fragile X syndrome will be warranted.

### Targeting proteins regulated by FMRP

#### Matrix metalloproteinase 9

Matrix metalloproteinases (MMPs) are a family of extracellular proteases that are involved in synaptogenesis, neurotransmission and synaptic plasticity (Ethell and Ethell, 2007; Wright and Harding, 2009; Huntley, 2012). MPP9 mRNA is among the putative mRNA targets of FMRP with increased translation in the absence of FMRP (Janusz et al., 2013). MMP9 is necessary for the development of fragile X phenotypes. Genetic deletion of MMP9 rescued key features of fragile X syndrome, including dendritic spine abnormalities, exaggerated mGluR-LTD, aberrant cognitive and social behaviors as well as macroorchidism in the mouse model (Sidhu et al., 2014). Minocycline, a tetracycline analogue with anti-inflammatory and antiapoptotic activity, has been used to inhibit MMP9 (Bilousova et al., 2009; Siller and Broadie, 2012). Treatment of young mice with minocycline increased phosphorylation and subsequent membrane insertion of AMPA GluR1 receptors (Imbesi et al., 2008). Minocycline rescued maturation of dendritic spines in the hippocampus thereby correcting the spine phenotype in *Fmr1* knockout mice (Bilousova et al., 2009). In addition, chronic minocycline reduced anxiety-related behavior, improved cognitive function in young *Fmr1* knockout mice (Bilousova et al., 2009), and reversed impaired social communication during mating among fragile X mice (Rotschafer et al., 2012). In comparison of the effect of minocycline on young and adult fragile X mice, it was found that minocycline could reduce locomotor activity in both young and adult mice, some behavioral improvements could be long-lasting in young mice, but not in adults. In addition, minocycline reduced audiogenic seizure susceptibility in young mice (Dansie et al., 2013). This study provides further evidence that minocycline can produce long-lasting benefits in the fragile X animal model.

Clinical trials have been conducted to evaluate the safety and efficacy of minocycline. An open-label add-on pilot trial demonstrated that minocycline is well tolerated and can provide significant functional benefits to fragile X patients (Paribello et al., 2010). In a controlled clinical trial, minocycline treatment was observed to lower the elevated plasma activity of MMP9 in individuals with fragile X syndrome. In some cases, changes in MMP9 activity were found to be positively associated with improvement in clinical measures (Dziembowska et al., 2013). In another randomized, double-blind, placebo-controlled and crossover trial, minocycline treatment administered for 3 months was found to be safe and produce greater global improvement than a placebo in children with fragile X syndrome (Leigh et al., 2013). However, longer trials are warranted to further assess the benefits and side effects related to minocycline.

#### p21-activated kinase

The small GTPase Rac1 and its effector p-21 activated kinase (PAK) are critical for regulation of actin polymerization, dendritic spine morphogenesis and synaptic plasticity (Hayashi et al., 2004; Zhang et al., 2005; Kreis and Barnier, 2009; Murata and Constantine-Paton, 2013). In hippocampal synapses of *Fmr1* knockout mice, physiological activation of the Rac-PAK signaling pathway is impaired (Chen et al., 2010). FMRP directly interacts with PAK1. Inhibition of PAK activity by expression of the dominant negative PAK (dnPAK) transgene results in a dendritic spine phenotype opposite to that of fragile X syndrome (Hayashi et al., 2007). A genetic expression of dnPAK in *Fmr1* knockout mice at least partially corrected abnormalities in spine length and density in the cortex, and fully restored deficits in cortical LTP. Additionally, several behavioral abnormalities including hyperactivity, stereotypy, anxiety, and deficits in trace fear memory were ameliorated (Hayashi et al., 2007). This genetic rescue of fragile X phenotypes in the mouse model suggests that the PAK signaling pathway could be a novel intervention site for fragile X syndrome and autism. Pharmacological treatment with the PAK inhibitor FRAX486 reversed dendritic spine phenotypes, and rescued seizures and behavioral abnormalities such as hyperactivity and repetitive movements in *Fmr1* knockout mice, further supporting PAK as a therapeutic target. Importantly, these effects could be achieved in adult *Fmr1* knockout mice with a single administration of FRAX486, demonstrating the potential for therapy in adults with fragile X syndrome (Dolan et al., 2013).

#### Amyloid precursor protein

Amyloid precursor protein (APP) is a transmembrane protein that plays roles in synaptogenesis and synaptic plasticity (Gralle and Ferreira, 2007; Randall et al., 2010; Nalivaeva and Turner, 2013). APP is translated upon mGluR5 activation. FMRP binds to APP mRNA and controls its translation (Westmark and Malter, 2007). Absence of FMRP leads to APP overexpression and diminished mGluR-induced synthesis (Westmark and Malter, 2007). Cleavage of APP can produce β-amyloid (Aβ), which is overexpressed in the brain of *Fmr1* knockout mice, suggesting a pathogenic role in fragile X syndrome (Westmark et al., 2010). Genetic reduction of APP/Aβ could partially or completely correct characteristic fragile X phenotypes, including audiogenic seizures, anxiety, dendritic spine morphology and exaggerated mGluR-LTD (Westmark et al., 2011), suggesting drugs directed at reducing Aβ in Alzheimer disease such as the secretase inhibitors or β-site APP cleaving enzyme (BACE-1) inhibitors may be applicable to fragile X syndrome (Malter et al., 2010; Westmark, 2013; Westmark et al., 2013).

#### Striatal-enriched protein tyrosine phosphatase

STEP, a brain-specific protein tyrosine phosphatase, dephosphorylates key signaling proteins including ERK1/2, p38 MAPK, the tyrosine kinase Fyn, and surface AMPA and NMDA receptors, thereby inactivating the kinases or promoting endocytosis of the receptors and opposing development of synaptic strengthening (Braithwaite et al., 2006; Goebel-Goody et al., 2012b). STEP is translated upon mGluR5 activation and mediates AMPA receptor internalization during mGluR-LTD (Zhang et al., 2008; Goebel-Goody and Lombroso, 2012). FMRP interacts with the transcript encoding STEP. The basal level of STEP is elevated and mGluR-dependent STEP synthesis is absent in *Fmr1* knockout mice (Goebel-Goody et al., 2012a). It is possible that the synaptic deficits and behavioral abnormalities in fragile X syndrome may be linked to the dysregulation of STEP (Goebel-Goody and Lombroso, 2012). Genetic reduction of STEP could attenuate audiogenic seizures, improve characteristic social abnormalities, and reverse anxiety-related behaviors in *Fmr1* knockout mice, suggesting that STEP might be a therapeutic target for treating fragile X patients (Goebel-Goody et al., 2012a).

#### Potassium channels

Potassium channels control the resting membrane potential and modulate the action potential waveform, mediating homeostasis of neuronal excitability (Misonou, 2010; Lee and Jan, 2012; Kim and Kaczmarek, 2014). In fragile X animal models, defects have been found in several types of potassium channels, such as the sodium-activated potassium Slack (Brown et al., 2010; Zhang et al., 2012), voltage-gated potassium channels Kv3.1b (Strumbos et al., 2010) and Kv4.2 (Gross et al., 2011; Lee et al., 2011), and large-conductance calcium-activated potassium (BK) channels (Deng et al., 2013; Zhang et al., 2014b).

FMRP interacts directly with Slack channels to enhance the channel activity, raising the possibility that Slack-FMRP interaction may link patterns of neuronal firing with changes in protein translation (Brown et al., 2010; Zhang et al., 2012). Disturbance of this link when FMRP is absent may underlie altered neuronal networks in fragile X syndrome. FMRP binds the mRNAs encoding Kv3.1b and is required for rapid experience-dependent regulation of Kv3.1b (Strumbos et al., 2010). FMRP also regulates mRNA translation and protein expression of Kv4.2; absence of FMRP-mediated control of Kv4.2 might contribute to excess neuronal excitability in *Fmr1* KO mice (Gross et al., 2011). Recovery of Kv4.2 after NMDA receptor-mediated degradation also requires FMRP, probably due to NMDA receptor activation-induced FMRP dephosphorylation, which turns off FMRP suppression of Kv4.2 (Lee et al., 2011). Significantly, treating hippocampal slices of *fmr1* KO mice with Kv4 channel blocker HpTx2 restored LTP induced by moderate stimuli, suggesting that potassium channels as an FMRP target could be of potential relevance to fragile X therapy (Lee et al., 2011). FMRP modulates action potential duration via its interaction with beta4 subunits of BK channels, thus regulating neurotransmitter release and synaptic transmission. Loss of these FMRP functions might be responsible for dysregulation of synaptic transmission in fragile X syndrome (Deng et al., 2013). Interestingly, a recent study showed that a selective BK channel opener (BMS-204352) could rescue multiple behavioral impairments (social, emotional and cognitive) in fragile X mice (Hebert et al., 2014), further demonstrating that potassium channels may open up new opportunities for treating fragile X syndrome.

In summary, multiple targeted pharmacological treatments have been found to rescue the phenotypes of fragile X animal models, but few have been beneficial to patients. Acamprosate, lovastatin, lithium and minocycline are the drugs that can be currently prescribed and have shown benefits to patients with fragile X syndrome. However, each single drug may not be effective for all patients. The combination of different drug therapies, together with behavioral interventions, will be necessary for better efficacy in treating fragile X syndrome (Braat and Kooy, 2014; Hagerman et al., 2014; Hagerman and Polussa, 2015).

## Targeted pharmacotherapy for Rett syndrome

RTT is a postnatal neurodevelopmental disorder that occurs mainly in females and is the leading cause of intellectual disability in this gender (Hagberg et al., 1983; Laurvick et al., 2006; Neul et al., 2010). Clinical presentation occurs in stages with an initial developmental stagnation that is followed by a rapid regression during which RTT individuals have autistic features, stereotypic hand movements, loss of language, and develop aggressive behaviors (Chahrour and Zoghbi, 2007; Percy et al., 2010). RTT girls also have seizures during childhood, daytime breathing arrythmias, dysautonomia, and develop scoliosis at later stages, which also include Parkinsonian features and loss of mobility (Glaze et al., 2010; Neul et al., 2014).

Loss-of-function mutations in the X-linked gene *MECP2* (methyl-CpG-binding protein-2) are responsible for RTT (Amir et al., 1999). In 95% of classic RTT cases, *MECP2* mutations occur *de novo* in germ cells and are usually on the paternal side (Girard et al., 2001; Trappe et al., 2001; Bienvenu and Chelly, 2006). There is a spectrum of mutations ranging from missense, nonsense, and frameshift mutations (Moretti and Zoghbi, 2006). RTT individuals appear to have a typical development until 6–18 months of age, when they start missing developmental milestones. The *MECP2* gene encodes a predominantly nuclear protein that is expressed ubiquitously but with predominance in the brain (Klose and Bird, 2006). The expression of *MECP2* follows a temporal pattern: it peaks in the early postnatal period when maturation of neurons and activity dependent refinement of synapses occurs (LaSalle et al., 2001; Shahbazian et al., 2002; Cohen et al., 2011). Its expression remains elevated during adulthood, when it is involved in the maintenance of existing neurons and synapses. MeCP2 was initially identified as a transcriptional repressor through its binding to methylated CpG sites in gene promoters (Nan et al., 1998; Jung et al., 2003), although it regulates gene expression through multiple mechanisms (Guy et al., 2011).

**Figure 2:**
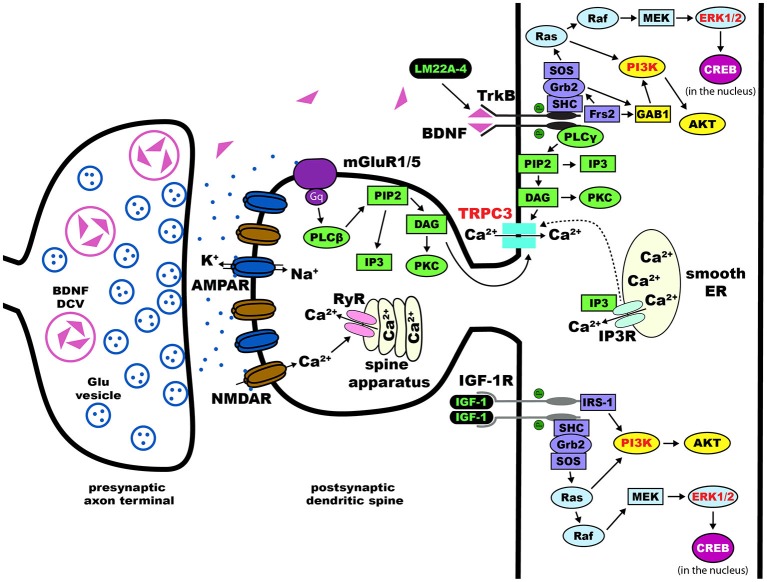
**Dendritic spines in Rett syndrome (RTT)**. Proposed intracellular mechanisms that mediate the effects of BDNF/TrkB on dendritic spine density and morphology. Trk receptors are activated upon binding of neurotophic factors, leading to dimerization and auto-phosphorylation. This process allows for the intracellular binding of adaptor proteins to Trk and activation of major pathways including Ras/ERK, PI3K, and PLCγ. Components of each of these three pathways have been implicated in the effects of BDNF on dendritic spines (highlighted in red text). Potential therapies for the treatment of RTT act on these pathways (highlighted in green text)—LM22A-4 directly phosphorylates TrkB and [1,3]IGF-1 activates the PI3K and Ras/ERK pathways. Abbreviations: AKT(PKB), protein kinase B; AMPAR, α-amino-3-hydroxy-5-methyl-4-isoxazolepropionic acid receptor; BDNF, brain-derived neurotrophic factor; CaMK, Ca2+/calmodulin-dependent protein kinase; cAMP, cyclic adenosine monophosphate; CREB, cAMP response element-binding protein; DAG, diacylglycerol; Frs2, fibroblast growth factor receptor substrate 2; GAB1, GRB2-associated-binding protein 1; Grb2, growth factor receptor-binding protein 2; IGF-1, insulin-like growth factor 1; IGF-1R, IGF-1 receptor; IP3, inositol triphosphate; MAPK, mitogen-activated protein kinase; MEK, MAPK//ERK kinase; NMDAR, *N*-methyl-D-aspartate receptor; PI3K, phosphoinositide 3-kinase; PIP2, phospohatidylinositol 4, 5 bisphosphate; PKC, protein kinase C; PLCγ, phospholipase C-γ; Raf, proto-oncogenic serine/threonine protein kinase; Ras, rat sarcoma proto-oncogenic G-protein; SHC, SH-2-containing protein; SH-2, src homology domain 2; SOS, nucleotide exchange factor son of sevenless; TrkB, tropomyosin related kinase B receptor; TRPC, transient receptor potential channel.

From multiple studies using different knockout mouse models, converging data indicates that the pathological deficit in RTT is at the microcircuit level involving the structure and function of synapses that are critical for synaptic transmission and plasticity (Chen et al., 2001; Guy et al., 2001, 2007; Figure 2). Therefore, most therapeutic treatments revolve around the restoration of synaptic function and maturation (Gadalla et al., 2011). Current preclinical research on therapeutic strategies and human clinical trials can be divided into drugs that target the primary cause, i.e., loss-of-function mutations in *MECP2*, and drugs that target downstream consequences, including neurotransmitter receptor systems, neurotrophins, and their intracellular signaling pathways (Tables 3, 4). Since this review is focused on pharmacological treatments, genetic manipulations will not be discussed here and readers are directed to recent reviews on this topic (Cobb et al., 2010; Gadalla et al., 2011; Guy et al., 2011). Treatment paradigms that have targeted pathways downstream of the *Mecp2* gene in mice, and the current human clinical trials are discussed in detail below.

**Table 3:** **Current preclinical trials in *Mecp2* deficient mice**.

Agent or Approach	Sponsor	Action
**BDNF boosters**
LM22A-4 (BDNF loop mimetic)	Sigma-Aldrich; RSRT	Partial agonist of TrkB receptors
		Improves breathing
RP103 (cysteamine)	Raptor Pharmaceuticals	Increases BDNF expression and release
		Improves breathing
**Serotonin agonists**
Sarizotan	Newron	5HT1 agonist
	Pharmaceuticals	Improves breathing
NLX-101	Neurolixis	5HT1 agonist
		Improves breathing
**NMDA receptors**
GluN2A negative allosteric modulator	Mnemosyne Pharmaceuticals	Improved visual cortical functioning
Ketamine	Approved drug	Non-competitive NMDA receptor antagonist
		Improved respiratory and locomotor function
**Antidepressants**
REV-003	Revive	Atypical antidepressant
(Tianeptine)	Therapeutics	Improves breathing
**MECP2 gene**
Read-through compounds	Research labs	Skip premature STOP codon in nonsense mutations

**Table 4:** **Current clinical trials in RTT individuals**.

Agent	Results	Trial outcome	Sponsor	Type
**Growth Factors**
Full length IGF-1	Phase 2	Safety confirmed on-going; scheduled through Fall 2015	Approved drug in children with growth failure	Double-blind; placebo controlled; injection
NNZ-2566 [1–3]IGF-1 analogue	Phase 2	Safety confirmed short-term trial positive	Neuren pharmaceuticals	Double-blind; placebo controlled; oral
**BDNF Effectors**
Copaxone	Phase 2	Improved ambulation	Approved drug	Open-label; injection
Fingolimod	Phase 1	On-going; scheduled through August 2017	Novartis pharmaceuticals; Approved drug-adults	Open-label; oral
**NMDA Antagonist**
Dextromethorphan	Phase 2	On-going; scheduled through June 2015	Approved drug in cough suppressant	Double-blind; placebo controlled
**Norepinephrine Effector**
Desipramine	Phase 2	On-going; scheduled through December 2014	Approved drug in Europe	Double-blind; placebo controlled
**Mitochondrial Effector**
EPI-743	Phase 2	Safety confirmed Improved head circumference growth	Edison pharmaceuticals	Double-blind; placebo controlled; oral

### Growth and neurotrophic factors

#### Brain-derived neurotrophic factor

*Bdnf*, the gene encoding the BDNF protein, is one of the first recognized direct targets of MeCP2 transcriptional regulation (Chen et al., 2003; Martinowich et al., 2003). BDNF binds to the high affinity receptor tropomyosin-related kinase B (TrkB), which activates intracellular signaling cascades critical for neuronal development, synaptic maturation, and learning and memory, like PLCγ-IP3R, PI3K-Akt, and MAPK-CREB (Segal and Greenberg, 1996). *Bdnf* expression is controlled by MeCP2 through complex interactions (Chen et al., 2003; Chang et al., 2006; Li and Pozzo-Miller, 2014), and reduced levels of BDNF mRNA and protein are considered to contribute to the pathophysiological mechanisms of RTT disease progression (Katz, 2014). Over-expression of BDNF in excitatory forebrain neurons of *Mecp2* deficient mice improved their RTT-like neurological phenotypes (Chang et al., 2006). Over-expression of *BDNF* rescued dendritic atrophy caused by shRNA-mediated *Mecp2* knockdown in cultured hippocampal neurons (Chapleau et al., 2009). The main limitation of recombinant BDNF is its low blood-brain barrier permeability, which prompted the search for BDNF “boosters” or mimetics with sufficient bioavailability in brain.

Currently, there are two clinical trials in RTT individuals testing compounds that boost BDNF levels: a Phase-1 open label trial of Fingolimod, and a Phase-2 open label trial of glatiramer acetate (Copaxone), both FDA-aproved drugs for the treatment of multiple sclerosis. Fingolimod is a modulator of the sphingosine-1 phosphate receptor, which leads to an increase in BDNF expression and activation of TrkB downstream signaling pathways (Deogracias et al., 2012). Glatiramer acetate is an immunomodulatory agent based on the amino acid structure of myelin basic protein (MBP) that is currently used for the treatment of relapsing-remitting multiple sclerosis. One of the proposed mechanisms of action of Copaxone is the increased expression and release of BDNF by autoreactive T-cells (Ziemssen et al., 2002).

Additonal potential leads with preclinical evidence include ampakines, which are known to increase BDNF expression by their action on AMPA-type glutamate receptors (Lauterborn et al., 2003). Peripheral treatment with ampakines significantly improved respiratory dysfunction in male *Mecp2* knockout mice (Ogier et al., 2007) that model the recurrent apneas suffered by RTT girls. Cysteamine and its dimer cystamine increase BDNF levels (Borrell-Pagès et al., 2006), which supports the Phase-2/3 clinical trial of RP103 for Huntington’s disease. More recently, a small molecule BDNF loop mimetic (LM22A-4) designed *in silico* to interact with the BDNF binding pocket in the TrkB receptor (Massa et al., 2010), restored respiratory regularity in female *Mecp2* heterozygous mice (Schmid et al., 2012; Kron et al., 2014).

#### Insulin Growth factor-1

Insulin Growth factor-1 (IGF-1) is a growth factor that, by binding to the IGF-1 receptor, activates similar intracellular signaling cascades to those triggered by BDNF activation of TrkB receptors. Indeed, IGF-1 modulates synaptic plasticity and neuronal maturation through a tyrosine kinase signaling pathway that includes PI3K-Akt and MAPK (Zheng and Quirion, 2004). Unlike BDNF, IGF-1 permeability through the blood brain barrier makes it an attractive compound for therapy. Intraperitoneal injection of the active IGF-1 tripeptide (also known as [1–3]IGF-1, or Glypromate) in male *Mecp2* knockout mice improved survival, locomotor activity, as well as social and anxiety behaviors (Tropea et al., 2009). Full-length IGF-1 (Mecasermin) is already approved by the FDA for the treatment of growth failure in children, and it was shown to have similar effects in male *Mecp2* knockout mice, as well as in female *Mecp2* heterozygous mice (Castro et al., 2014); a cautionary note is that full-length IGF-1 can worsen the metabolic syndrome of *Mecp2* deficient mice (Pitcher et al., 2013). The effects of full-length IGF-1 are due to the direct activation of IGF-1 receptors and its downstream signaling cascades, while the effects of the [1–3]IGF-1 tripeptide may reflect increased expression of IGF-1 (Corvin et al., 2012), although its molecular mechanism-of-action is currently unknown. Based on these promising leads, a Phase-2 double-blinded placebo-controlled clinical trail is underway to treat 3–10 years old RTT patients with full-length IGF-1 (Khwaja et al., 2014). More recently, a Phase-2 double-blinded placebo-controlled clinical trail in 16–45 years old RTT individuals has been initiated to test the efficacy of NNZ-2566, a protease-resistant analogue of the [1–3]IGF-1 tripeptide.

### Neurotransmitter systems

#### Monoamines

Multiple studies have documented low levels of monoamine markers (dopamine, 5-HT, noradrenaline) in autopsy RTT brains and in *Mecp2* deficient mice (Brücke et al., 1987; Lekman et al., 1989; Roux et al., 2008). Desipramine is an antidepressant that blocks the uptake of noradrenaline, and has been shown to reverse the depletion of tyrosine hydroxylase in the brainstem, helping with the regulation of breathing and extending the life-span of male *Mecp2* knockout mice (Roux et al., 2007; Zanella et al., 2008). Desipramine is currently in a Phase-2 double blinded, placebo-controlled clinical trial for RTT. The atypical tricyclic antidepressant tianeptine (REV-003) also improved respiratory activity in *Mecp2* deficient mice, although this effect may reflect modulation of monoamine levels, glutamate receptor function, or BDNF levels (McEwen et al., 2010). Recently, preclinical studies in *Mecp2* deficient mice have demonstrated that the 5-HT1a agonist Sarizotan inhibited expiratory neuron activity, which significantly improved breathing patterns and reduced the frequency of apneas (Abdala et al., 2014).

#### Glutamate

The NMDA-type of glutamate receptors is altered in *Mecp2* knockout mice (Blue et al., 1999, 2011; Maliszewska-Cyna et al., 2010). Dextromethorphan is a NMDA receptor antagonist that has been tried in an open label clinical trial without any significant benefit. The FDA-approved NMDA receptor antagonist ketamine has been useful in *Mecp2* knockout mice to improve RTT-like phenotypes (Kron et al., 2012); based on these encouraging results, a clinical trial of low dose ketamine is currently planned. A delay in the developmental switch in the expression of GluN2 subunits of the NMDA receptor in the visual cortex contributes to visual acuity deficits in *Mecp2* deficient mice, which were improved by genetic deletion of the GluN2A subunit (Durand et al., 2012); a negative allosteric modulator selective for GluN2A-containing NMDA receptors is currently in preclinical trials in *Mecp2* deficient mice.

#### GABA

Several studies have shown that GABAergic signaling is impaired in *Mecp2* deficient mice, which alters the excitatory/inhibitory balance. Impaired GABAergic inhibition was described in the brainstem (Medrihan et al., 2008), thalamus (Zhang et al., 2010) and hippocampus (Calfa et al., 2015), and involved impaired evoked and spontaneous inhibitory synaptic transmission and numbers of GABAergic synapses on principal neurons. Furthermore, selective deletion of *Mecp2* in GABAergic neurons led to impaired GABAergic transmission, cortical hyperexcitability and several neurological features of RTT and ASDs (Chao et al., 2010). Also, deletion of *Mecp2* specifically in excitatory neurons caused impaired GABAergic transmission on cortical pyramidal neurons, which led to seizures and cortical hyperexcitation (Zhang et al., 2014a). A preclinical study in female *Mecp2* heterozygous mice demonstrated that increasing ambient GABA levels by inhibiting the GABA reuptake transporter improved respiratory patterns (Abdala et al., 2010). Vigabatrin is an antiepileptic drug that irreversibly inhibits GABA transaminase, inhibits GABA catabolism and thereby increases GABA levels (Connelly, 1993). The drug is already FDA approved for use in epilepsy syndromes. Planning for a clinical trial for RTT is underway. However, retinal toxicity may limit the chronic use of this medication.

### Mitochondrial function

RTT is associated with high levels of systemic oxidative stress and alteration in mitochondrial morphology, while plasma levels of oxidative stress biomarkers correlate with disease severity and progression (De Felice et al., 2012). Based on these observations, the small molecule EPI-743 was tested in a phase-2 placebo controlled trial of RTT individuals. Results from this exploratory trial revealed improvement in head growth but not in other core features of RTT. The structure of EPI-743 is based on vitamin E and its proposed mechanisms-of-action includes augmenting glutathione synthesis and acting at the mitochondrial level to regulate electron transport.

### “Read-through” compounds to increase MECP2 expression

In approximately one-third of RTT individuals, a nonsense mutation in *MECP2* (e.g., R168X, R255X) leads to the premature termination of transcripton due to a premature STOP codon (Schanen et al., 2004; Percy et al., 2007). Clinically, these RTT individuals have a more severe presentation than RTT individuals with missense *MECP2* mutations that result in single amino acid substitutions. Aminoglycoside antibiotics like gentamycin are so-called “read-through” compounds because they allow ribosomal read-through of the premature STOP codon during translation, yielding a full-length functional protein. Aminoglycoside and non-aminoglycoside “read-through” compounds have been tested for therapeutic efficacy in Duchenne muscular dystrophy and cystic fibrosis (Zingman et al., 2007). Preclinical studies relevant to RTT have demonstrated that either gentamycin or geneticin were effective in translating a full-length functional MeCP2 protein in a lymphocyte cell line derived from an individual with a R255X nonsense mutation (Popescu et al., 2010), and in transgenic mice expressing a R168X nonsense mutation (Brendel et al., 2011). Furthermore, gentamycin increased dendritic spine density in neurons derived from induced pluripotent stem cells (iPSC) that were obtained from a RTT individual with a Q244X nonsense mutation (Marchetto et al., 2010). However, the renal and auditory toxicity of gentamycin has limited its progress toward human clinical trials and prompted the development of new “read-through” compounds with better safety profiles.

## Open questions and challenges

### Design and quality of preclinical studies

Although many targeted treatments have shown efficacy across multiple aspects in ASD animal models, none has thus far demonstrated the same effectiveness in patients (Katz et al., 2012; Hagerman et al., 2014). Hurdles to translational success may include lack of rigorous standards in assessing the effect of treatment, lack of transparency in reporting preclinical data, as well as publication bias caused by disregarding negative results in preclinical studies (Katz et al., 2012). The translational success stories in fragile X and Rett syndromes indicates that the effectiveness of targeted treatments in animal models can turn into clinical efficacy in humans, provided that studies are well designed, the models are reliable and robust, and that preclinical outcome measures are relevant to patients (Anagnostou, 2012; Lipton and Sahin, 2013; Castro et al., 2014; Khwaja et al., 2014). To achieve more translational success in ASDs, efforts are needed to improve the quality of preclinical studies in the future. Strict standards must be implemented for preclinical study designs and result reporting to ensure that clinical trials are grounded on reliable preclinical data (Katz et al., 2012; Landis et al., 2012).

One of the key issues in preclinical studies is that almost every study has applied its own animal behavioral experiment battery to evaluate the effects of genetic or pharmacological manipulations, making comparison of efficacy among different treatments a challenge. This situation will be greatly improved if animal behavioral test batteries for identifying potential therapy could be standardized. Despite the discrepancies in test batteries from different laboratories, many of the targeted treatments achieve predicted effect and rescue at least part of phenotypic features of the disorders in animal models (Braat and Kooy, 2014; Hagerman et al., 2014).

### Challenges in clinical trials

Despite progress made in clinical trials, one of the major concerns for these studies is the lack of appropriate outcome measures for an objective assessment of patients’ daily performance (Berry-Kravis et al., 2011; Wijetunge et al., 2013; Berry-Kravis, 2014; Braat and Kooy, 2014; Jacquemont et al., 2014). As a result, some improvements following therapeutic intervention might not be observed without the right measurements. It is helpful that researchers have been trying to formulate suitable outcome measures, which could be adjusted accordingly for patients (Berry-Kravis et al., 2013), such as quantitative event related potential and expressive language outcome measure which have been developed for fragile X patients (Hessl et al., 2009). It is anticipated that these measures will better demonstrate improvements in language and cognition in individual patients. In addition, molecular markers, such as ERK, BDNF and MMP9, can be measured to provide a direct biochemical evidence of improvement with targeted treatments (Hagerman et al., 2014).

Differential responses to one specific treatment have been observed in individuals with ASDs. Some subgroups within the patient cohorts respond to the therapy where others do not, as evident from clinical trials in fragile X syndrome (Jacquemont et al., 2011; Berry-Kravis et al., 2012). It is therefore important and helpful to develop biomarkers for selection of patients that will benefit from a specific therapy depending on their genotypes and/or neurobiological phenotypes. For example, if follow-up studies would confirm that AFQ056 treatment significantly improves behavior in fully methylated patients, detecting the methylation status of *Fmr1* promoter might help identify a subgroup of fragile X patients that will respond well to this treatment (Jacquemont et al., 2011; Braat and Kooy, 2014).

Interference with the molecular pathways disturbed in ASDs has led to the initiation of clinical trials. The fragile X and Rett syndromes are the prototypes of neurodevelopmental disorders for which targeted treatments are becoming realities (Samaco and Neul, 2011; Chapleau et al., 2013a,b; Braat and Kooy, 2014). However, it is unlikely that a single compound will be effective in all individuals with ASDs. Thus, it will be necessary to test combinations of multiple drugs that each rescue part of clinical presentations to find a combination of drugs that works more efficiently than each on its own. Importantly, pharmaceutical interventions need to be paired with appropriate behavioral and cognitive training to maximize the efficacy. This represents a significant step towards more personalized approaches to treating fragile X, RTT and other ASDs in future, with a better chance of success (Castro et al., 2013; Wijetunge et al., 2013; Braat and Kooy, 2014; Hagerman et al., 2014).

### The timing of treatment initiation

It is generally believed that earlier interventions in developmental disorders will have better outcomes. Abnormalities occurring during early development have usually been considered irreversible in adulthood. However, studies in mouse models of neurodevelopmental disorders, including fragile X and Rett syndromes, suggest that many pathophysiological aspects associated with the disorders can be reversed by genetic or pharmacological manipulations performed during adulthood (Castrén et al., 2012). Improvements have also been observed in adult patients with fragile X syndrome treated with arbaclofen and AFQ056 (Jacquemont et al., 2011; Henderson et al., 2012). These findings suggest that the pathophysiology associated with the loss of FMRP or MeCP2 may not arise from irreversible developmental brain abnormalities, but result from functional disturbances of neural circuits that could be corrected in adulthood, providing a potential rational basis for treatment of neurodevelopmental disorders in adulthood (Castrén et al., 2012; Wijetunge et al., 2013; Hagerman et al., 2014). Despite these exciting advances, translation from animal experimentation to clinical practice and finding out the right initiation timing for treating individual patients will be challenging issues in future investigation (Castrén et al., 2012; Wijetunge et al., 2013).

## Conclusions

The increasing need for effective treatments of fragile X and Rett syndromes, coupled with the availability of animal models and iPSC-derived neurons from human individuals with these disorders (Marchetto et al., 2010; Wang and Doering, 2012), has promoted translational studies towards identifying potential therapeutics. New opportunities are now emerging that might lead to development of novel pharmacotherapies for patients with fragile X and Rett syndromes. The development of mechanism-based targeted treatments will require more extensive multidisciplinary researches (Chapleau et al., 2013a; Berry-Kravis, 2014; Braat and Kooy, 2014; Hagerman et al., 2014). It is the responsibility of the research community to rigorously validate disease models, outcome measures and study designs that will produce robust and reproducible preclinical findings with clear relevance to human diseases (Katz et al., 2012; Landis et al., 2012).

The rational therapeutics for ASDs requires the knowledge of an entire spectrum of symptoms that relate to each specific disorder. The complicated pathophysiology of fragile X and Rett syndromes should be taken fully into account in designing preclinical and clinical studies. It should be recognized that loss of FMRP or MeCP2 will affect a number of downstream targets which exist not only on neurons, but on glial cells and other tissues (McCauley et al., 2011; Cheng et al., 2012; Derecki et al., 2013; Ausió et al., 2014); that the loss can have direct, as well as indirect and cumulative effects on development and function of the central nervous system or other organs, leading to distinct phenotypic consequences that possibly require different treatment strategies. Understanding these complexities will be essential for selecting relative therapeutics for patients (Chapleau et al., 2013a,b; Delorme et al., 2013; Berry-Kravis, 2014; Hagerman et al., 2014). It is inspiring to see that fragile X and Rett syndromes are becoming role models for how research in animal models could be translated into patients and how management of symptoms of the diseases could extend and improve the quality of life, providing further insights into understanding and treating ASDs and other neurodevelopmental diseases.

## Conflict of interest statement

The authors declare that the research was conducted in the absence of any commercial or financial relationships that could be construed as a potential conflict of interest.

## Footnotes

^1^www.clinicaltrials.gov

^2^www.fragilex.org

^3^www.clinicaltrials.gov

^4^www.clinicaltrials.gov
